# Occupation and Environmental Heat-Associated Deaths in Maricopa County, Arizona: A Case-Control Study

**DOI:** 10.1371/journal.pone.0062596

**Published:** 2013-05-29

**Authors:** Diana B. Petitti, Sharon L. Harlan, Gerardo Chowell-Puente, Darren Ruddell

**Affiliations:** 1 Department of Biomedical Informatics, Arizona State University, Tempe, Arizona, United States of America; 2 School of Human Evolution and Social Change, Arizona State University, Tempe, Arizona, United States of America; 3 Spatial Sciences Institute, University of Southern California, Los Angeles, California, United States of America; University of Hong Kong, Hong Kong

## Abstract

**Background:**

Prior research shows that work in agriculture and construction/extraction occupations increases the risk of environmental heat-associated death.

**Purpose:**

To assess the risk of environmental heat-associated death by occupation.

**Methods:**

This was a case-control study. Cases were heat-caused and heat-related deaths occurring from May-October during the period 2002–2009 in Maricopa County, Arizona. Controls were selected at random from non-heat-associated deaths during the same period in Maricopa County. Information on occupation, age, sex, and race-ethnicity was obtained from death certificates. Logistic regression analysis was used to estimate odds ratios for heat-associated death.

**Results:**

There were 444 cases of heat-associated deaths in adults (18+ years) and 925 adult controls. Of heat-associated deaths, 332 (75%) occurred in men; a construction/extraction or agriculture occupation was described on the death certificate in 115 (35%) of these men. In men, the age-adjusted odds ratios for heat-associated death were 2.32 (95% confidence interval 1.55, 3.48) in association with construction/extraction and 3.50 (95% confidence interval 1.94, 6.32) in association with agriculture occupations. The odds ratio for heat-associated death was 10.17 (95% confidence interval 5.38, 19.23) in men with unknown occupation. In women, the age-adjusted odds ratio for heat-associated death was 6.32 (95% confidence interval 1.48, 27.08) in association with unknown occupation. Men age 65 years and older in agriculture occupations were at especially high risk of heat-associated death.

**Conclusion:**

The occurrence of environmental heat-associated death in men in agriculture and construction/extraction occupations in a setting with predictable periods of high summer temperatures presents opportunities for prevention.

## Introduction

Published reports suggest that agricultural workers are at elevated risk of death due to heat-related illness [1], [2]. Prior research also shows that work in construction [3] and extraction (mining) occupations [4], [5] is associated with an increased risk of heat-related illness. More generally, engaging in heavy labor [6], work outdoors that involves exposure to hot and humid environments [1], and heavy exertion in athletes [7] and military trainees [8], [9] puts individuals at risk for heat-related illness and death.

The Maricopa County Department of Public Health (MCDPH) attempts to systematically ascertain environmental heat-caused and heat-related deaths that occur in the county during the summer. Maricopa County, Arizona is a setting with predictable conditions of high heat in the summer, and deaths associated with high environmental heat continue to occur in spite of the predictability of high heat. Information from this surveillance effort provided a unique opportunity to estimate the magnitude of the increase in the risk of heat-associated death among people with occupations that often involve work outdoors.

A study of farm workers in North Carolina showed that a high proportion (94%) of Hispanic farm workers reported working in extreme heat [10]. Based on this observation, we conducted an analysis to assess whether the relative risk of heat-associated death among people in agriculture occupations was higher in Hispanics than in non-Hispanic whites.

## Methods

### Overview of Study

This was an epidemiologic case-control study. The source of data was death certificates. Cases were deaths occurring in May through October during the period January 1, 2002-December 31, 2009 in Maricopa County that were identified as heat-caused or heat-related by the MCDPH as part of its program of on-going surveillance of heat-associated deaths. Controls were deaths occurring in Maricopa County selected at random from among all non-heat associated deaths in Maricopa County during the same period with frequency matching of cases and controls by year of death using a case-control ratio of 1∶2. Two controls were selected per case to increase statistical power and make estimates of odds ratios more precise.

### Ethics Review

The study was reviewed and approved by both the Arizona State University Institutional Review Board and the Arizona Department of Health Services Human Subjects Review Board. The research used information recorded on death certificates for individuals identified by the MCDPHS and the Medical Examiner as heat-associated. Patient data from hospital records were used by MCDPH to identify heat-associated deaths. The use by the MCDPH of patient data from hospital records is a component of its routine public health activity in conducting surveillance to monitor heat-associated health events. Data from hospital records were used by the Maricopa County Medical examiner to certify deaths possibly due to heat as heat-associated for the purposes of completing the death certificate. The use of data from hospital records by the Medical Examiner without consent is permitted by law. In Arizona, hospitalized patients are not asked either to approve or disapprove the use of information from their hospital records for research. The Arizona Department of Health Services Human Subjects Review Board waived the requirement for consent based on a judgment that the research posed no more than minimal risk; that the rights and welfare of human subjects were not adversely affected by the waiver; and that obtaining consent from people who have died is impracticable. The Arizona State University Institutional Review Board did not require consent because it does not consider research using information about people who have died to be human subjects research.

### Description of the MCDPH Surveillance System

Since 2006 prospectively and retrospectively since 2000, environmental heat-associated deaths have been identified by the MCDPH in a formal surveillance system. The MCDPH begins its surveillance process by gathering information on suspected heat-associated deaths using a variety of sources. These include the Office of Medical Examiner case list, which identifies suspected heat-associated deaths referred to the Medical Examiner; the Arizona Department of Health Services, which identifies deaths with environmental heat mentioned on death certificates; local hospitals, which identify suspected heat-associated morbidity/mortality cases reported by emergency department staff and infection staff; Humans Remains Release Forms, which include death reports received from hospitals and other institutions within 24 hours of death; and media reports of heat deaths. To assure that the MCDPH list of heat-associated deaths is complete and that no deaths have been erroneously certified as heat-associated by community physicians rather than the Medical Examiner, a multiple-causes-of-death search is done daily using the electronic death certificate database. The search is done to identify both International Classification of Disease, Tenth Revision (ICD-10) codes associated with environmental heat (X30-Exposure to excessive natural heat; T67.x-Effects of Heat and Light; and P81.0-Environmental hyperthermia of newborn) and key phrases in the text fields for causes and underlying causes of death (heat exposure; environ; exhaustion; sun; heat stress; heat stroke; hyperthermia).

Suspected cases of heat-associated deaths identified by the MCDPH surveillance system that do not appear on the Medical Examiner's case list as suspected heat-associated deaths are referred to the Medical Examiner. The Medical Examiner either rules out or confirms the deaths as environmental heat-associated deaths based on information about the circumstances of death and his/her examination of the remains when indicated. Certification by the Maricopa County Medical Examiner is the basis for designation of death as being environmental heat-associated and the certified environmental heat-associated deaths include not only deaths caused by environmental heat exposure but also those in which environmental heat exposure precipitated a death technically caused by something else (e.g., an arrhythmia). The MCDPH heat-associated death surveillance system thus differs from most other heat mortality surveillance systems, which include only deaths for which the underlying cause of death appears as environmental heat exposure using the ICD-10 codes listed above.

Suspected heat-associated deaths identified in the MCDPH surveillance system certified by the Maricopa County Medical Examiner are classified in one of two categories. Deaths in the first category are directly caused by environmental heat exposure. In these deaths, environmental heat is mentioned in Part I of the death certificate (diseases or conditions in the direct sequence causing death) whether or not environmental heat is described as the underlying cause of death on Part I of the death certificate. Deaths classified in the second category – heat-related deaths – mention environmental heat terms in Part II of the death certificate causes of death (diseases and conditions contributing but not directly resulting in the death) sequence but not in the Part I sequence. The current study includes both categories – heat-caused and heat-related – and labels them heat-associated deaths in keeping with the terminology used by the MCDPH in its surveillance operation. For the purposes of surveillance and in this study, a death is considered environmental heat-associated if it was related to heat generated by the climate (e.g., sun, humidity, etc.) and excludes heat from man-made sources such as ovens or manufacturing equipment.

### Classification of Occupation

Text information recorded in the industry and occupation fields of the standard death certificate was inspected and used to classify the occupation of cases and controls. A person was classified as having a construction/extraction occupation if the term “construction” or “mining” or “miner” appeared in either the industry or the occupation text field on the death certificate or if the description of the person's occupation or industry is included in the Standard Occupation Classification major group 47-0000 – Construction and Extraction Occupations [11]. A person was classified as having an agricultural occupation if “agriculture,” “landscaping,” or “nurseryman” appeared in the text field of the death certificate or if the description of their occupation or industry is included in the Standard Occupation Classification major group 45-0000– Farming, Fishing and Forestry Occupations [12]. A person was classified with an occupation of homemaker if “homemaker” or “housewife” appeared in the occupation or industry text field of the death certificate. A person was classified as having an unknown occupation if “unknown” appeared both in the industry and the occupation fields or if both fields were blank. All other people were classified as having “other known occupation.” The latter category included people who were described as having never worked.

Classification of occupation was completed by one investigator (Petitti) blind to whether the death was heat-associated. A formal assessment of the reliability of the classification of occupation was completed. In it, an investigator not involved in the initial classification (Harlan) classified occupation in a random sample of 150 deaths blinded to case-control status and to initial classification. Cohen's Kappa was used to assess reliability [13]. It was 0.97 (95% confidence interval 0.93, 1.00).

### Race/Ethnicity

Information on race/ethnicity was obtained from the ethnicity and race fields of the death certificates. The coding of race and ethnicity on the Arizona death certificate uses categories and follows rules specified by the National Center for Health Statistics for the United States Standard death certificate. Over the period of this study, changes were made in the level of detail requested for the ethnicity field and the completeness of designation of individuals as Hispanic or non-Hispanic in the ethnicity field changed. In an attempt to make the data on race/ethnicity consistent across time, race/ethnicity was classified in a single category. A person was classified as Hispanic if the term “Hispanic” appeared in the ethnicity field of the death certificate. People designated as non-Hispanic and those not designated as Hispanic were further sub-classified as non-Hispanic white, African-American, Native American, or Asian/Pacific Islander based on text information in the race field of the death certificate.

### Analysis

Cases and controls were first described using percent distributions. Unconditional multiple logistic regression was used to estimate age-adjusted odds ratios for heat-associated death and 95% confidence intervals according to occupation. The percentage of deaths with an unknown occupation was substantially higher in cases than in controls. For this reason, the referent for the analysis of occupation was chosen to be other known occupation. Seventy-five percent of heat-associated deaths were in men and all but four of the subjects classified as having a construction/extraction or agriculture occupation were men. For this reason, the multiple logistic regression analyses to estimate the odds ratios for heat-associated death were done separately in men and women.

To assess whether the association between occupation and heat-associated death was modified by race/ethnicity or age, odds ratios for heat-associated deaths were estimated according to occupation in strata of race-ethnicity and age. Because the number of heat-associated deaths in African-American and Native American men was small, these analyses were limited to non-Hispanic white and Hispanic men.

The Hosmer Lemeshow chi-square statistic was used to assess model fit [14]. Presented models showed no evidence of lack of fit based on a P value greater than 0.05. In all presented models, confidence intervals for odds ratios that excluded 1.00 were statistically significant using a P≤0.05 to define statistical significance and thus P values are not shown. All analyses were done using SAS^®^ software version 9.2 [15].

## Results

There were 463 cases of heat-associated death during the period 2002–2009; 925 controls age 18+ years of age were selected for these cases. Nineteen cases of heat-associated death with known age less than 18 years of age were excluded. Nine cases of heat-associated death with unknown age were included in the analysis after noting that all of them had an entry in the occupation and industry field whereas approximately 98% of deaths at ages less than 18 had blank entries for occupation and industry. There were 444 cases and 925 controls included in the analysis.

Table 1 shows the number and percentage distribution of occupation, race/ethnicity and age for cases and controls overall and in men and women separately. Of the 444 heat-associated deaths, 332 (75%) were in men. Of the heat-associated deaths in men, 115 (35%) had construction/extraction or agriculture occupations. Among the 324 men with known age; 231 (71%) were age 18–64 years.

**Table 1:** Number and percent distribution of cases and controls by occupation, race/ethnicity, and age for all subjects and in men and women separately.

Characteristic	Sub-characteristic	All Subjects		Men		Women	
		Cases N = 444	Controls N = 925	Cases N = 332	Controls N = 465	Cases N = 112	Controls N = 460
		N (%)	N (%)	N (%)	N (%)	N (%)	N (%)
Occupation	Construction/Extraction	76 (17.1)	68 (7.4)	76 (22.9)	68 (14.6)	0 (0.0)	0 (0.0)
	Agriculture	40 (9.0)	27 (2.9)	39 (11.8)	24 (5.2)	1 (0.9)	3 (0.7)
	Homemaker	67 (15.1)	179 (19.4)	0 (0.0)	0 (0.0)	67 (59.8)	179 (38.9)
	Unknown	118 (26.6)	16 (1.7)	80 (24.1)	13 (2.8)	38 (33.9)	3 (0.7)
	All Other Known	143 (32.2)	635 (68.7)	137 (41.3)	360 (77.4)	6 (5.4)	275 (59.8)
Race/Ethnicity	Non-Hispanic White	263 (59.2)	788 (85.2)	191 (57.5)	389 (83.7)	72 (64.3)	399 (86.7)
	Hispanic	126 (28.4)	97 (10.5)	101 (30.4)	55 (11.8)	25 (22.3)	42 (9.1)
	Native American	22 (5.0)	16 (1.7)	18 (5.4)	10 (2.2)	4(3.6)	6 (1.3)
	African American	27 (6.1)	23 (10.5)	18 (5.4)	11 (2.4)	9 (8.0)	12 (2.6)
	Asian/Pacific Islander/Unknown	6 (1.4)	1 (0.1)	4 (1.2)	0 (0.0)	2 (1.8)	1 (0.2)
Age in Years	18–34	43 (9.7)	41 (4.4)	38 (11.4)	26 (5.6)	5 (4.5)	15 (3.3)
	35–49	112 (25.2)	62 (6.7)	93 (28.0)	34 (7.3)	19 (17.0)	28 (6.1)
	50–64	114 (25.7)	156 (16.9)	100 (30.1)	100 (21.5)	14 (12.5)	56 (12.2)
	65–74	67 (15.1)	164 (17.7)	47 (14.2)	96 (20.7)	20 (17.9)	68 (14.8)
	75–84	63 (14.2)	238 (25.7)	32 (9.6)	112 (24.1)	31 (27.7)	126 (27.4)
	85+	37 (8.3)	264 (28.5)	14 (4.2)	97 (20.9)	23 (20.5)	167 (36.3)
	Unknown	8 (1.8)	0 (0.0)	8 (2.4)	0 (0.0)	0 (0.0)	0 (0.0)

Some percentages do not sum to 100.0 because of rounding.

Table 2 and Table 3 show age-adjusted odds ratios and 95% confidence intervals for heat-associated death by occupation and race/ethnicity in men and women separately. In men, with all known occupations as the reference category, the age-adjusted odds ratios for heat-associated death were 2.32 (95% confidence interval 1.55, 3.48) in association with construction/extraction occupations, 3.50 (95% confidence interval 1.94, 6.32) in association with agriculture occupations, and 10.17 (95% confidence interval 5.38, 19.23) in association with an unknown occupation. Using non-Hispanic white men as the reference category, the age-adjusted odds ratios for heat-associated death were significantly increased in Hispanic men (odds ratio 2.69; 95% confidence interval 1.79, 4.05) and Native-American men (odds ratio 2.43; 95% confidence interval 1.04, 5.65). Simultaneous adjustment for age, occupation and race-ethnicity did not change by much the odds ratios. Simultaneous adjustment attenuated the odds ratio for heat-associated death in Hispanics because of the strong correlation between occupation and Hispanic race/ethnicity. In men, 62% of those in agriculture occupations (39/63) were Hispanic.

**Table 2:** Adjusted odds ratios and 95% confidence intervals for heat-related death by occupation in men and women.

Occupation	Adjusted Only for Age				Adjusted for Age and Race Ethnicity			
	Men		Women		Men		Women	
	Odds Ratio	(95% CI)	Odds Ratio	(95% CI)	Odds Ratio	(95% CI)	Odds Ratio	(95% CI)
Construction/Extraction	2.32	(1.55, 3.48)	*	–	2.04	(1.34, 3.11)	*	–
Agriculture	3.50	(1.94, 6.32)	*	–	2.83	(1.48, 5.43)	*	–
Homemaker	*	–	0.86	(0.55, 1.37)	*	–	0.75	(0.46, 1.20)
Unknown	10.17	(5.38,19.23)	6.32	(1.48,27.08)	9.83	(5.16,18.72)	6.08	(1.34, 27.51)
All Other Known	1.00	referent	1.00		1.00	referent	1.00	referent

* Cannot be estimated because of small numbers.

CI Confidence Interval.

**Table 3:** Adjusted odds ratios and 95% confidence intervals for heat-related death by race/ethnicity in men and women.

Race/Ethnicity	Adjusted Only for Age				Adjusted for Age and Occupation			
	Men		Women		Men		Women	
Non-Hispanic White	1.00	referent	1.00	referent	1.00	referent	1.00	referent
Hispanic	2.69	(1.79, 4.05)	2.79	(1.56, 5.00)	1.84	(1.17, 2.91)	2.96	(1.63, 5.40)
Native American	2.43	(1.04, 5.65)	3.81	(1.51, 9.57)	2.02	(0.85, 4.81)	2.82	(0.73,10.91)
African American	2.22	(0.99, 4.99)	2.62	(0.68,10.07)	1.79	(0.75, 4.29)	3.81	(1.49, 9.73)
Asian/Pacific Islander/Unknown	*	*	*	*	*	–	*	–

* Cannot be estimated because of small numbers.

CI Confidence Interval.

In women, using all other known occupation as the reference category, the age-adjusted odds ratio for heat-associated death was 6.32 (95% confidence interval 1.48, 27.08) in association with an unknown occupation. Using non-Hispanic white women as the reference category, the age-adjusted odds ratios for heat-associated death were significantly increased in Hispanic women (odds ratio 2.79; 95% confidence interval 1.56, 5.00) and Native-American women (odds ratio 3.81; 95% confidence interval 1.51, 9.57) and increased, but not significantly, in African-American women (odds ratio 2.62; (95% confidence interval 0.68, 10.07).

To assess whether race and age modified the associations of occupation with heat-associated death, we did stratified analyses. These analyses excluded Native Americans and African-Americans and those with unknown or Asian race/ethnicity because the numbers of cases and controls in these groups was small.

Table 4 shows age-adjusted odds ratios for heat-associated death by occupation separately in white non-Hispanic and Hispanic men. As in the other analyses, all other known occupation was used as the reference category. The age-adjusted odds ratio for heat-associated death was increased in association with construction/extraction occupations only in the non-Hispanic white men (odds ratio 2.10; 95% confidence interval 1.26, 3.50). Age-adjusted odds ratios for heat-associated death in association with agriculture occupations were increased by 2–3 both in non-Hispanic white and Hispanic men. Only the increase in non-Hispanic white men was statistically significant (95% confidence interval 1.01, 9.88) but the confidence intervals in both groups were wide and overlapped. The odds ratios for heat-associated death in association with having an unknown occupation were increased in both non-Hispanic white (odds ratio 10.45; 95% confidence interval 4.73, 23.05) and Hispanic (odds ratio 5.58; 95% confidence interval 1.67, 18.67) men. The increases were statistically significant in both groups but the 95% confidence intervals were wide and overlapped.

**Table 4:** Number and percent distribution of cases and controls and age-adjusted odds ratios and 95% confidence intervals for heat-associated death by occupation in non-Hispanic white and Hispanic men.

Occupation	Non-Hispanic White				Hispanic			
	Cases N = 191	Controls N = 389	Age-Adjusted		Cases N = 101	Controls N = 55	Age-Adjusted	
	N (%)	N (%)	Odds Ratio	95% CI	N (%)	N (%)	Odds Ratio	95% CI
Construction/Extraction	43 (22.5)	51 (13.1)	2.10	(1.26, 3.50)	19 (18.8)	12 (21.8)	1.20	(0.46, 3.11)
Agriculture	9 (4.7)	9 (2.3)	3.16	(1.01, 9.88)	26 (25.7)	13 (23.6)	2.04	(0.80, 5.20)
Unknown	40 (51.8)	9 (2.3)	10.45	(4.73,23.05)	30 (29.7)	4 (7.3)	5.58	(1.67,18.67)
All Other Known	99 (20.9)	320 (82.3)	1.00	referent	26 (25.7)	26 (47.3)	1.00	referent

Some percentages do not sum to 100.0 because of rounding.

CI Confidence Interval.

Table 5 shows the number and percentage distribution of cases and controls, odds ratios, and 95% confidence intervals for heat-associated death by occupation in non-Hispanic white and Hispanic men in strata of age –18–49 years, 50–64 years, 65–84 years, and 85+ years. Data are shown unadjusted and adjusting for race/ethnicity. The data are sparse in some age groups. The unadjusted odds ratio for heat-associated death in association with construction occupation was 1.32 (95% confidence interval 0.58, 3.03) in men 18–49 years. Adjustment for race/ethnicity did not change this estimate by much (odds ratio 1.38; 95% confidence interval 0.60, 3.22). The unadjusted odds ratios for heat-associated death in association with construction occupation were 2.94 for men age 50–64 years, 1.62 for men 65–84 years and 7.40 for men 85+ years. Adjustment for race/ethnicity did not change these odds ratios by much.

**Table 5:** Number and percent distribution of cases and controls and unadjusted and adjusted* odds ratios and 95% confidence intervals for heat-associated death by occupation in non-Hispanic white and Hispanic men by age.

Age	Occupation	Cases	Controls	Unadjusted		Adjusted for Race/Ethnicity *	
		N (%)	N (%)	Odds Ratio	95% CI	Odds Ratio	95% CI
Age 18-49 Years	Construction/Extraction	27 (24.8)	14 (31.8)	1.32	(0.58, 3.03)	1.39	(0.60, 3.22)
	Agriculture	21 (19.3)	5 (11.4)	2.88	(0.95, 8.70)	3.35	(1.02,11.00)
	Unknown	26 (23.9)	1 (2.8)	17.83	(2.26,140.41)	19.60	(2.44,157.61)
	All Other Known	35 (32.1)	24 (54.6)	–	referent	–	referent
Age 50-64 Years	Construction/Extraction	14 (19.7)	10 (12.1)	2.94	(1.17, 7.35)	2.66	(1.04, 6.76)
	Agriculture	4 (5.6)	3 (3.6)	2.80	(0.59,13.26)	1.60	(0.30, 8.69)
	Unknown	22 (31.0)	5 (6.0)	9.23	(3.19,26.66)	8.54	(2.93,24.90)
	All Other Known	31 (43.7)	65 (78.3)	–	referent	–	referent
Age 65-84 Years	Construction/Extraction	10 (15.2)	25 (13.5)	1.62	(0.72, 3.67)	1.53	(0.66, 3.53)
	Agriculture	3 (9.1)	6 (3.2)	4.05	(1.24,13.29)	2.03	(0.54, 7.56)
	Unknown	13 (19.7)	4 (2.2)	13.17	(4.06,42.72)	10.83	(3.24,36.16)
	All Other Known	37 (56.1)	150 (81.1)	–	referent	–	referent
Age 85+ Years	Construction/Extraction	3 (30.0)	10 (11.1)	7.40	(1.31,41.79)	7.25	(1.28,41.12)
	Agriculture	4(40.0)	6 (6.7)	16.45	(2.97,91.16)	14.27	(2.36,86.42)
	Unknown	0(0.0)	0 (0.0)	**	–	**	–
	All Other Known	3(30.0)	74 (82.2)	–	referent	–	referent

Some percentages do not sum to 100.0 because of rounding.

* Hispanic compared with non-Hispanic white.

** cannot be estimated due to small numbers.

CI Confidence Interval.

The odds ratios for heat-associated death in association with agriculture occupations were increased in all age groups. However, the odds ratios in association with agriculture occupations in men age 65–84 years and 85+ years were somewhat higher than at younger ages: 4.05 (95% confidence interval 1.24, 13.29) and 16.45 (95% confidence interval 2.97, 91.16) respectively. Adjustment for race/ethnicity (Hispanic versus non-Hispanic white) in these age-strata attenuated the association between heat-associated death and occupation because of the strong correlation between being Hispanic and agriculture occupation.

## Discussion

Our data show an association of heat-associated death with construction/extraction and agriculture occupations in men in Maricopa County, Arizona as expected given prior research. The increase in the risk of heat-associated death in association with agriculture occupations was not greater in Hispanic than in non-Hispanic white men. However, Hispanic men were more likely to work in agriculture and the difference in the distribution of occupation in Hispanic men contributed to, but does not fully explain, the overall higher risk of heat-associated death among Hispanic men in this study. Our study was not able to disentangle fully the independent effects on heat-associated death of race/ethnicity and occupation because of the small number of deaths in some categories. The study does not rule out the possibility that men who were African-American and Native Americans might be at especially high risk of heat-associated death.

Our study suggests that the increase in the risk of heat-associated death in association with construction/extraction and agriculture occupations may be modified by age. In men age 18–49 years, the risk of heat-associated death was close to 1.00 and not statistically significantly increased in association with construction occupations. In men age 65 or more years, the magnitude of the increase in the risk of heat-associated death in association with agriculture occupations was large: about 4 in men age 65–84 years and about 7 in men age 85+ years. These findings were based on only a small number of cases of heat-associated deaths in older men and must be interpreted cautiously.

Our findings are consistent with published reports from prior research that examined death certificate data for heat-associated deaths among workers in occupations classified as agriculture, construction, and mining (extraction) [1]–[6]. Work in agriculture and construction occupations is primarily out of doors during the day time and often involves heavy exertion. In its 2010 annual report on heat-associated deaths, the MCDPH noted that 71% of heat-associated deaths in that year occurred out of doors [16]. Heavy exertion increases the risk of heat-associated illness in military trainees [8], [9] and athletes [7].

A 2008 report from the Centers for Disease Control and Prevention estimated that heat-related average annual death rate for agricultural workers was 0.39 per 100,000, compared with 0.02 for all United States civilian workers [1]. The Bureau of Labor Statistics reported that the number of workers in the construction and extraction occupations (Standard Occupational Codes 47-0000) in the Phoenix-Mesa Metropolitan Statistical Area was 114,570 in 2002 [17], 130,120 in 2005 [18], and 96,390 in 2009 [19]. The number of workers in agricultural occupations (Standard Occupational Codes 45-0000) reported by the Bureau of Labor Statistics was 3,410 in 2002 [17], 2,610 in 2005 [18], and 3,480 in 2009 [19]. These numbers underestimate the number of workers in these occupations because self-employed people and undocumented workers are not counted. For this reason, an accurate estimate of the absolute rate of heat-associated death for construction and agriculture occupations in Maricopa County cannot be derived. The data from the Bureau of Labor Statistics suggest that a substantial number of workers in Maricopa County are at risk of the adverse effects of high environmental heat.

We found a very high risk of heat-associated death in associated with having an unknown occupation both in men and women. Unknown occupation is a marker for homelessness. Other investigations of heat-associated death conducted by the Arizona Department of Health Services and the MCDPH have identified homelessness as an important contributor to heat-associated death in Maricopa County [20] and elsewhere in Arizona [21].

Investigation of heat-associated death conducted by the Arizona Department of Health Services has identified attempts to cross the desert to enter the United States illegally as a factor in heat-associated death in Arizona [21]. Information about occupation available for these people is often very limited. The higher risk of heat-associated death in association with having an unknown occupation that we report is probably also explained in part by the lack of information on occupation for these deaths.

Our study has several limitations. A first major concern is the use of death certificates as the source of information on occupation. The accuracy of this information is uncertain. More importantly, our study does not directly assess the effect of work related to occupation in construction and agriculture. In fact, only 12 of the heat-associated deaths were designated as being deaths due to injury at work. All of these deaths were in men; seven were in men with a construction/extraction or agriculture occupation and 2 were in men with unknown occupation. Guidelines to Medical Examiners and Coroners on how to complete the “injury at work” section of the death certificate specify that a death should be designated as due to an injury at work if the injury occurred: while working or in vocational training on job premises, while on break or at lunch or in parking lot on job premises, while working for pay or compensation, including at home; while working as a volunteer law enforcement official, or while traveling on business, including to/from business contacts [22]. It is not certain whether heat-associated illness that occurs because of work is viewed universally as due to an injury. Some deaths designated by the Medical Examiner as heat-associated might occur at home after becoming ill from heat exposure at the workplace.

The fact that the highest risk of heat-associated death in association with agriculture occupations was observed in men age 65 years or more raises the possibility that occupation may be a marker for the risk of heat-associated death and not due directly to work in agriculture or work out of doors. Determining whether there was a direct link between work in construction/extraction or agriculture and death due to heat would require collection of information directly from observers. Further research based on more detailed information about the circumstances of death for heat-associated deaths in those with a construction or agriculture occupation might clarify the contribution of work in a hot environment to the death.

Recognizing the problem of heat-related illness and death in workers, California and Washington have adopted stringent workplace safety standards designed to prevent heat-related illness [23], [24]. California first adopted the workplace safety standards on an emergency basis in 2005 and made them permanent in 2006. The initial standard required employers to: 1) provide fresh water so that each employee can drink one quart per hour and encourage them to drink frequently; 2) provide access to shade and encourage employees to take a cool down rest in the shade for 5 minutes; 3) train all employees and supervisors about heat-related illness; and 4) develop and implement written procedures for complying with the heat illness prevention standard. In 2010, California strengthened its heat illness prevention standard by requiring employers in five industries – agriculture, construction, landscaping, oil and gas extraction, and transportation/delivery of agricultural products – to implement specific other procedures when temperatures reach 95 degrees Fahrenheit. These are requirements to: 1) ensure effective communication so employees can contact their supervisor when necessary; 2) observe employees for alertness and signs or symptoms of heat illness; 3) remind employees throughout the work shift to drink plenty of water; and 4) closely supervise a new employee for the first 14 days of employment.

Washington State has applied the standards to all employers with employees in outdoor settings [24]. It is similar to the initial California standard and requires that: 1) employers address outdoor heat exposure in a written accident prevention plan; 2) employers provide drinking water; 3) certain specific actions be taken in response to signs and symptoms of heat-related illness, and 4) workers and supervisor undergo training about heat-related illness.

In 2011, the Occupational Health and Safety Administration (OSHA) initiated a nationwide outreach campaign to raise awareness among workers and employers about the hazards of working outdoors in hot weather [25]. The campaign focuses on four measures to prevent heat-related illness in outdoor workers: drink water often, rest in the shade, report symptoms early, and know what to do in an emergency. The effect of this standard on heat-related illness in workers has not been evaluated.

One of the challenges of better understanding heat-related illness and mortality is determining the threshold of human tolerance to excessive temperatures. The Centers for Disease Control and Prevention (CDC) defines sustained periods of hot weather as those with daytime temperatures of 105 or more degrees Fahrenheit (40.6 degrees Centigrade) and a nighttime minimum temperature of 80 or more degrees Fahrenheit (26.7 degrees Centigrade) persisting for at least 48 hours [1], [26]–[27]. The CDC links sustained periods of hot weather by this definition with adverse health effects. In Maricopa County, during the period 2002–2009, there was an annual average of 7 episodes of sustained hot weather as defined by the CDC (unpublished data); the shortest period was 6 days (in 2004) and the longest was 21 days (in 2007);. For the period encompassed in this study, 2002–2009, the daily maximum temperature reached or exceeded 110 degrees Fahrenheit (43.3 degrees Centigrade) an average of 24 days each year (unpublished data). The persistent occurrence of heat-associated death in a setting that has such predictable periods of very hot weather is noteworthy. Given the predictability of high temperature during the summer in Maricopa County, heat-associated death in workers in the construction and agriculture is probably largely preventable. In the Abu Dhabi, United Arab Emirates, where summer ambient air temperatures often reach 135 degrees Fahrenheit (57.2 degrees Centigrade) or more with 90% humidity, a successful program aimed at reducing heat-related morbidity and mortality in workers subject to heat stress has been described [28].

Our findings may have larger implications. Periods of high temperature are projected to become more common [29], [30]. Occupationally related exertion is one of several factors that affect the risk heat-related illness and death [31]. Recognizing who is vulnerable to illness when temperatures are high provides information that can be used to target interventions and create adaptations that can be put in place proactively [32].
